# The role of the insulin-like growth factor-1 system in breast cancer

**DOI:** 10.1186/s12943-015-0291-7

**Published:** 2015-02-15

**Authors:** Panagiotis F Christopoulos, Pavlos Msaouel, Michael Koutsilieris

**Affiliations:** Department of Experimental Physiology, Medical School, National and Kapodistrian University of Athens, 75 Mikras Asias Street, 11527 Goudi, Athens, Greece; Department of Internal Medicine, Jacobi Medical Center, Albert Einstein College of Medicine, Bronx, NY USA

**Keywords:** Insulin-like growth factor-1 (IGF-1), IGF-1 receptor (IGF-1R), IGF-binding proteins (IGFBPs), IGF-1 signaling, IGF-1 regulation, Estrogens, Estrogen receptor (ER), Breast cancer, Mammary tumorigenesis

## Abstract

IGF-1 is a potent mitogen of major importance in the mammary gland. IGF-1 binding to the cognate receptor, IGF-1R, triggers a signaling cascade leading to proliferative and anti-apoptotic events. Although many of the relevant molecular pathways and intracellular cascades remain to be elucidated, a growing body of evidence points to the important role of the IGF-1 system in breast cancer development, progression and metastasis. IGF-1 is a point of convergence for major signaling pathways implicated in breast cancer growth. In this review, we provide an overview and concise update on the function and regulation of IGF-1 as well as the role it plays in breast malignancies.

## Introduction

The Insulin Like Growth Factor 1 (IGF-1) system which includes IGF-1, IGF-binding proteins (IGFBPs) and the IGF-1 receptor (IGF-1R), plays a significant role in human physiology, particularly in the development and function of many tissues, including the mammary gland. IGF-1 is a key mediator of mammary terminal end bud and ductal formation during development [1]. Experimental findings have demonstrated that normal rat mammary epithelial cells continue to proliferate in serum-free media in response to IGF-1, suggesting that the IGF-1 system plays an important role in mammary gland function and maintenance [2]*.* In contrast, over the last two decades many studies have implicated the IGF-1 system in the development of several malignancies including breast cancer. Indeed, breast tumors may aberrantly express each component of the IGF-1 system. To date, an increasing number of studies have attempted to elucidate the molecular mechanisms underlying the association of the IGF-1 system with breast malignancy [3-5].

### The IGF-1 system in breast cancer

IGF-1 is a 7.7 kDa single-chain polypeptide encoded by chromosome 12. The 70 amino acid IGF-1 protein consists of four domains [6] and is produced primarily in the liver under the direct stimulation of Growth Hormone (GH). *IGF-1* gene expression is controlled by both transcriptional and post-translational modifications. Distinct IGF-1-peptides may arise via the utilization of different promoters, alternative splicing, proteolytic processing and glycosylation events [7]. IGF-1 is expressed in almost every human tissue. In both normal mammary gland and malignant breast tissues, IGF-1 is mainly expressed by stromal and only rarely by epithelial cells [8]. Circulating IGF-1 levels vary depending on the person’s age: there is initially an increase in serum IGF-1 from birth to puberty, followed by a stable decline with age in response to the lower GH levels [9,10]. The mitogenic, anti-apoptotic and other effects of IGF-1 protein are mainly mediated by the transmembrane tyrosine kinase receptor IGF-1R, which in contrast to IGF-1, is mainly expressed in the mammary epithelium. Tetrameric IGF-1R consists of two α- identical and two β- identical subunits. Ligand binding and subsequent phosphorylation of IGF-1R triggers the activation of two major signaling cascades via the insulin receptor substrate 1 (IRS-1): the phosphatidylinositol 3-kinase/AKT kinase (PI3K/AKT) pathway and the RAF kinase/mitogen activated protein kinase (RAF/MAPK) pathway which stimulate proliferation and protection from apoptosis [11]. Notably, the IRS-1 has been found to be overexpressed in primary breast tumors [12]. The bioavailability and half-life of circulating IGF-1 is regulated by a family of six IGF-binding proteins (IGFBP1-6) [13]. Each IGFBP can bind to IGF-1 with high affinity and is regulated by several specific IGBPB proteases. Approximately 1% of circulating IGF-1 remains unbound, while the rest is mainly bound to IGFBP3, forming a complex with an acid-labile subunit [14].

Breast Cancer (BrCa) remains one of the leading causes of cancer-related death worldwide. The heterogeneity and variability in treatment and survival response, underscore the need to elucidate the biological mechanisms driving BrCa. A proposed molecular profile approach for breast tumor classification defines distinct molecular subtypes of the disease based on differences in the expression patterns of estrogen receptor (ER), progesterone receptor (PR) and HER2 (ERBB2) [15]. Although BrCa has been intensely studied and multiple reported biomarkers and molecular targets have been reported in the literature, only a few are of proven relevance to routine clinical practice. Both *in vitro* and *in vivo* models, as well as clinical and epidemiological data have indicated the role of the IGF-1 system in BrCa via many diverse endocrine, paracrine and autocrine signaling pathways [16,17]. Although some of these findings are conflicting, many components of the IGF-1 system are known to be altered during BrCa establishment and progression [9,16,18].

### Circulating IGF-1 levels and breast cancer (endocrine role)

Since the initial report by Peyrat et al. [19], many epidemiological and prospective studies have attempted to confirm the positive correlation between plasma IGF-1 levels and BrCa risk. A pooled data analysis of seventeen prospective studies from twelve countries by the *Endogenous Hormones and Breast Cancer Collaborative Group* showed a clear association between circulating IGF-1 and BrCa risk in Estrogen Receptor positive (ER^+^) tumors independent of IGFBP3 and menopausal status [20]. This finding is also supported by the data analysis from the *European Prospective Investigation into Cancer and Nutrition* cohort [21]. Serum IGF-1 levels have also been positively associated with increased disease risk among BRCA gene mutation carriers (hereditary BrCa) in an Italian cohort study [22]. In contrast no correlation was found between IGF-1 levels and breast cancer development in a cohort of Brazilian women [23] or women during early pregnancy [24]. Mammographic density is one of the strongest predictors of breast cancer development and may be associated with serum IGF-1 levels in premenopausal women. Dorio et al., found an association between mammographic density and serum IGF-1 in premenopausal women [25] though recent studies did not corroborate this finding [26,27].

The association of IGF-1 with disease prognosis following tumor establishment is also currently under investigation. High circulating IGF-1 levels have been positively correlated with bad prognosis in patients undergoing endocrine therapy [28], while another study found that high serum IGF-1 is associated with increased all-cause mortality in a cohort of women with established breast malignancy [29]. Further studies measuring both mRNA and protein levels are warranted, in order to better delineate the role of circulating IGF-1 in disease risk and progression.

### IGF-1 polymorphisms in breast cancer

Over the last decade, there has been increasing interest in the studying of the genomic analysis of the *IGF-1* gene for specific alterations involved in cancer formation and progression. One of the most studied genetic variations of *IGF-1* is a polymorphic sequence of repeating cytosine-adenine dinucleotides (CA) ranging from 10 to 24 repeats in length, with the CA_19_ being the most common allele. This repeating sequence is located almost 1 kb upstream of the transcription initiation site and is thus considered to be a promoter polymorphism likely implicated in regulating IGF-1 protein levels. No significant association has been found between CA_19_ and BrCa risk among Arab Omani women in both post- and pre-menopausal status [30]. In contrast, in another case control study among African-American and Hispanic women, a significant correlation between the non-19/non-19 allele polymorphisms and breast cancer was detected, predominantly in premenopausal women [31]. No association between CA 17, 19 and 20 alleles and breast cancer risk was found in a meta-analysis by Huang et al. [32], whereas a more recent meta-analysis of 11 studies by He et al. reported that CA 19/19 may confer a decreased risk for BrCa development in Caucasian but not in Asian women [33]. Indeed, there is growing evidence that the CA_19_ allele is associated with increased incidence of breast and other cancers in Asians [34]. Allelic length has also been found to correlate with disease development. BrCa risk is increased in Iranian women carrying two alleles of CA longer than 19 and decreased in those carrying two alleles shorter than 20 [35]. Other genetic variations including single nucleotide polymorphisms (SNP’s) and SNP combinations (haplotypes) have been studied for potential associations with BrCa. Most of the SNPs studied are in areas located in highly evolutionary conserved regions (ECR) near to the transcription factor binding domains (BD), thereby affecting transcription regulation. Biong et al. reported an association between a common IGF-1 genetic haplotype, plasma IGF-1 levels and mammographic density in postmenopausal Norwegian women [36] while neither a single SNP, nor any diplotype (combination of two haplotypes) was associated with circulating IGF-1 levels in a multivariate analysis of Swedish women. In the same study, a rare diplotype variant found in a small proportion of women (n = 14/325) strongly correlated with development of early-onset BrCa [37]. Another individual SNP (*rs 7965399*), located in the 5′-unstranslated region of IGF-1 gene, near the transcription initiation site, has been associated with BrCa risk in a recessive model, particularly in estrogen receptor negative (ER^−^) or early menopause Chinese women [38]. The *Breast and Prostate Cohort Consortium* (BPC3), a collaboration of large US and European Cohorts, genotyped a total of 1416 SNP’s for 24 genes involved in the IGF-1 pathway in 6,292 Caucasian postmenopausal women with diagnosed BrCa as compared to 8,135 controls and did not find any SNP associations with BrCa risk [39]. In addition other large collaborative studies using data from the BPC3, genotyped a total of 302 SNPs in a sample size of more than 5,500 Caucasian women and detected a clear association between genetic variations of IGF-1 and plasma IGF-1 levels but no association with BrCa risk [40]. Despite the large number of studies identifying IGF-1 gene polymorphisms in association with BrCa risk, only a few have investigated the relationship of such polymorphisms with disease progression. Homozygotes for the non-19/non-19 CA allele with non metastatic BrCa have been found to have favorable prognostic factors and longer disease-free and overall survival [41], while HER2+ patients carrying the rare SNP *rs2946834* allele have poorer prognosis and decreased event-free survival probably due to increased IGF-1 circulating levels [42]. Very recently, a meta-analysis of Genome-Wide Association studies (GWAS) found that the SNP *rs703556*, located 222 kb upstream of *IGF-1* gene, correlates with mammographic density [43]. The importance of this finding is highlighted by the fact that GWAS utilizes a less “biased” approach by first detecting, among a large pool of candidate SNPs, those SNPs that strongly correlate with disease risk or progression. GWAS then determine if these SNPs are located near to the *IGF-1* gene. This is in contrast to other studies described above, which use genetic variants of *IGF-1* as candidate risk factors.

### Autocrine and paracrine role of IGF-1 in breast cancer

In contrast to the serum IGF-1 expression findings described above, increased IGF-1 mRNA levels within tissue samples may confer a favorable outcome and have been associated with increased disease-free survival (DFS) in patients with diagnosed estrogen receptor positive (ER^+^) breast cancer [44]. This finding was further supported by microarray analysis of tumor samples revealing increased IGF-1 expression in a specific BrCa subtype associated with better prognosis [45]. The contradictory role of circulating and tissue IGF-1 may be partially explained by clinical data showing a lack of correlation between circulating and tissue IGF-1 levels [46].

Several factors could explain this discrepancy. Circulating IGF-1 levels may reflect the IGF-1 expression from several organs/tissues and/or metabolic processes and may thus not correlate with BrCa status *per se.* Thus, tissue IGF-1 levels may be a better marker of tumor IGF-1 expression compared to serum levels, as has already been established in mammary gland branching morphogenesis [47]. Within tissue microenvironment, increased IGF-1 levels may reflect cell differentiation into a less aggressive phenotype. Ethnic and other differences among the different groups studied may also account for these conflicting results. Further studies are warranted in order to delineate the importance of circulating (endocrine) versus tissue (autocrine/paracrine) levels of IGF-1 in disease risk and progression. Future investigation should focus on specific ethnic groups, measure IGF-1 levels in both serum and breast tissue of the same patient and correlate those findings with disease risk and outcome factors.

In addition to the above epidemiological findings, many pre-clinical laboratory studies have focused on the impact of IGF-1 in cancer cell proliferation, migration, tumor growth and metastasis using *in vitro* and *in vivo* models to identify the signaling pathways involved in these processes. Triple-negative cells (negative for PR, ER and HER2) have shown increased proliferation and survival in response to exogenous IGF-1 via both AKT and MAPK pathways [48]. IGF-1 release from differentiated or precursor adipocytes derived from obese patients was two fold higher compared to lean individuals. It also induced the proliferation of MCF7 cells in co-culture experiments, further supporting the notion that obesity *per se* could contribute to BrCa progression [49]. Microarray analysis in an *ex vivo* model of primary breast fibroblasts derived from BrCa patients revealed a signature of genes associated with proliferation following stimulation with IGF-1 [50]. An earlier study suggested that IGF-1-mediated stimulation of proliferation might act through transcriptional regulation [51]. Administration of IGF-1 induces invasion of MDA-MB-231 BrCa cells via the formation of cellular protrusions called lamellipodia, a characteristic projection at the front edge of motile cells believed to function as the motor pulling the cell forward during cell migration [52]. *In vivo* findings also indicate a tumor-promoting role of IGF-1. MCF-7 cells stably overexpressing IGF-1 induce significantly higher tumor volumes compared with control or mock cells in mouse xenografts [51]. It is also well known that autocrine IGF-1 signaling affects mammary development. Indeed, conditional and epithelial-specific knockout of IGF-1 results in reduced mammary branching during ductal growth [53]. Targeting IGF-1 in mammary epithelium will clarify the role of autocrine IGF-1 signaling in neoplastic transformation of breast epithelium. Future studies should focus on this approach.

**Figure 1:**
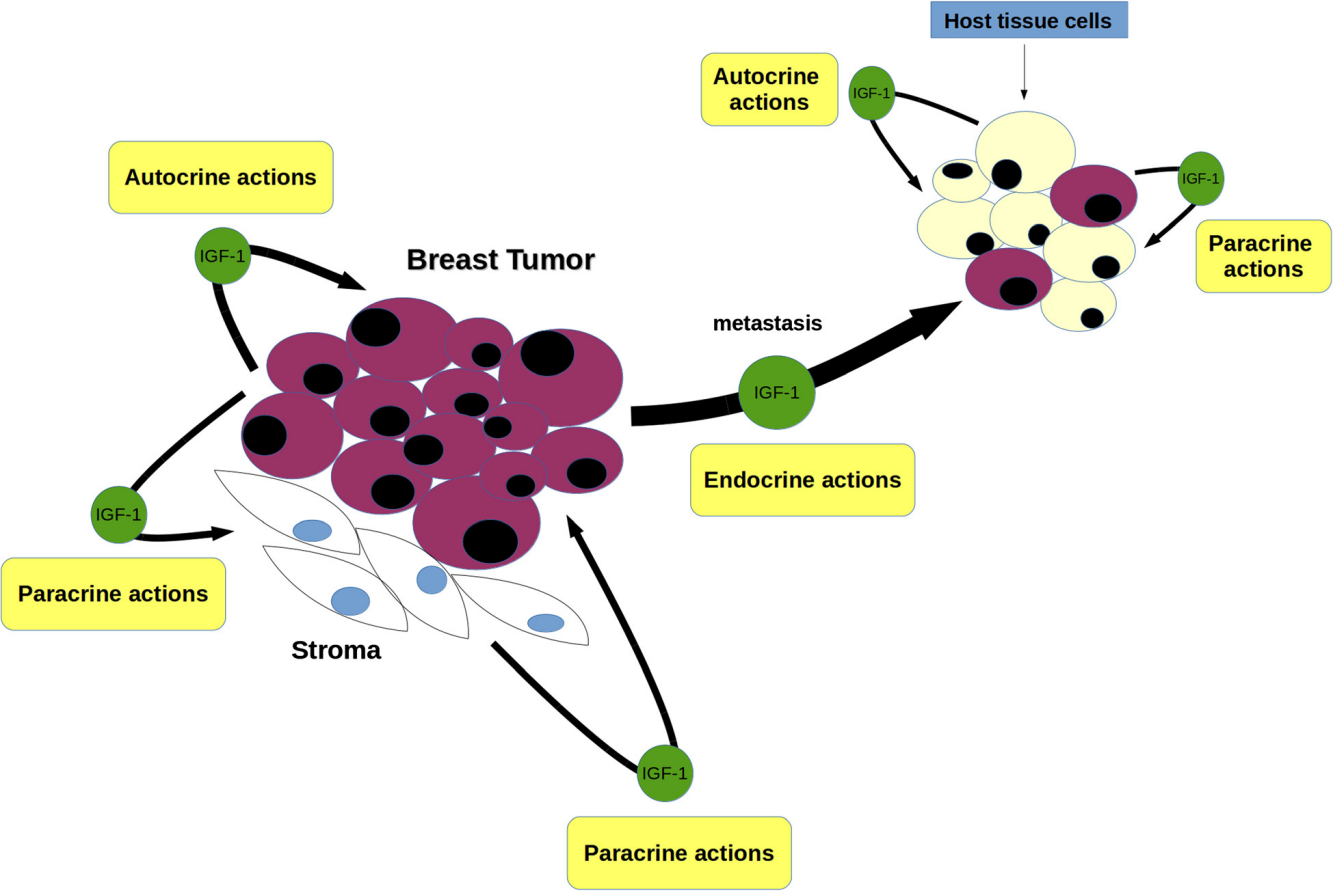
**The IGF-1 role in breast cancer.** IGF-1 is mostly secreted by stromal cells and acts through paracrine signaling to adjacent epithelial tumor cells and vice versa. Once the disease is well established within the primary breast tumor microenvironment, IGF-1 autocrine (within epithelium) and endocrine (via the systemic circulation) activity facilitates disease progression and metastasis respectively. Within the new tumor location, the interplay between metastasized breast cancer cells and host tissue cells, drive the last to a more cancerous phenotype via IGF-1 paracrine signaling. In addition, aberrant IGF-1 expression by host tissue cells supports BrCa proliferation via autocrine signaling.

Recent research efforts have used animal models to delineate the impact of IGF-1 in complex disease events including metabolic regulations, angiogenesis and metastasis. Prenatal administration of IGF-1 in pregnant wild type (WT) mice results in increased body weight, higher breast density with longer ductal elongation and higher breast stem/progenitor cell populations of prepubescent offspring compared to phosphate buffered saline (PBS) controls [54]. Transgenic mice specifically overexpressing IGF-1 in mammary epithelium demonstrate upregulation of the Vascular Endothelial Growth Factor (VEGF), a pro-angiogenic factor, in prepubertal glands and induction of cyclooxygenase-2 (COX-2), an inflammatory molecule that is also associated with angiogenesis and is responsible for formation of prostaglandin (PG), [55]. It is also well established that caloric restriction prevents mammary tumorigenesis in rodents. Mouse models have indicated that IGF-1 may play a key role in tumor reduction via the AKT/mTOR pathway following caloric restriction [56]. Furthermore, the effect of caloric restriction in IGF-1 levels may regulate luminal tumor growth by modulating the epithelial-mesenchymal transition (EMT) process and chemokine milieu [57]. This pathway appears to be involved in the metastasis of breast tumors. Bone is one of the most common distant target sites for BrCa metastasis. Hiraga et al. showed that bone derived IGF-1 stimulates proliferation and bone localization of breast cancer cells *in vivo* through activation of AKT and recruitment of transcription factor NF-kB [58]. The fact that IGF-1 is mostly expressed by stroma underscores the significance of the interplay between stroma and epithelium dururing IGF-1 paracrine signaling resulting in disease establishment and progression. The excess of paracrine IGF-1 signaling via stromal production may trigger epithelial IGF-1 expression, leading to a more malignant phenotype. Within the epithelium, aberrant IGF-1 autocrine signaling could further contribute to disease aggressiveness. Future studies should focus on clarification of the paracrine role of IGF-1, in establishment and progression of breast carcinoma, via *in vivo* models specifically overexpressing or lacking IGF-1 in stroma. In conclusion, IGF-1 plays a key role in BrCa development, progression and metastasis. It achieves this through autocrine, paracrine and endocrine interactions between stromal and breast cancer cells within many microenvironments: the primary tumor site, the circulation and/or host tissue and migrating tumor cells at metastatic sites (Figure 1).

### IGF-1 binding proteins (IGFBPs) in breast cancer

A wealth of experiment evidence implicates IGFBPs, in BrCa pathophysiology, particularly IGFBP_2_, IGFBP_3_ and IGFBP_5,_. Most studies suggest that elevated systemic or tissue IGFBP levels are found in breast malignancies. The interaction of IGFBPs with BrCa can be either inhibitory or stimulatory via both IGF-dependent and -independent pathways. Although many studies report a positive association between BrCa risk and circulating IGFBPs levels (mainly IGFBP_3_) calculated from IGF1/IGFBPs ratios, only a few studies have directly measured free molecules with conflicting results. Among Brazilian women, no association has been detected between serum IGFBP_3_ and BrCa risk [23], whereas a pooled data analysis of prospective studies revealed a positive (although non-statistically significant) correlation between IGFBP_3_ and BrCa [20]. Furthermore, plasma IGFBP_3_ levels have been shown to be independent of mammographic density, which is a well-established BrCa risk factor [26,27].

The link between IGFBPs and BrCa outcome is also unclear. Recent studies did not detect a significant association between plasma IGFBP_3_ and overall survival or risk of all-cause mortality [28,29]. Although other findings indicate that a poor BrCa outcome may be associated with systemic and tissue IGFBP_3_ [59-61], studies on the correlation between IGFBP_2_ levels and disease prognosis are limited. Hensch et al. reported an association of IGFBP_3_ with BrCa variables known to be predictive of outcomes, including tumor grade, body mass index (BMI), ER and premenopausal status. The same study reported a positive association between tissue IGFBP_2_ levels and overall survival, suggesting that protein levels of both IGFBPs in BrCa are under hormonal and obesity control [62]. In contrast, IGFBP_5_ tumor tissue levels have been found to be significantly associated with poor disease outcome and the addition of IGFBP_4_ (measured as IGFBP_5_/IGFBP_4_ ratio expression levels) further increased prognostic power [63].

At the DNA level, the genetic polymorphism *A-202C* of *IGFBP*_*3*_ (SNP, *rs2854744*) at the promoter region has been extensively studied for potential associations with circulating IGFBP_3_ levels and BrCa risk. Multiple studies have reported that the *A-202C* genetic variation of *IGFBP*_*3*_ is not associated with BrCa risk among Caucasian, African and Asian women [31,34,38,39], whereas others provide evidence for a correlation between *A-202C* polymorphism and increased IGFBP_3_ circulating levels. The postulated correlation is thought to be, due to enhanced promoter activity, indicating a regulation *in cis* [40,64]. Other SNPs of both IGFBP_1_ and IGFBP_3_ have also been associated with plasma IGFBP_3_ levels [36,64]. Additionally, a Mendelian randomization study showed that the a allele of *IGFBP*_*3*_ SNP *rs2854744* is associated with increased levels of circulating IGFBP_3_ and decreased BrCa risk [65].

Although the exact mechanism needs to be elucidated, *in vitro* experiments indicate that IGFBP_3_ inhibits BrCa proliferation and induces apoptosis. A recent study suggested that IGFBP_3_ regulates cell cycle by inducing a G1/S phase arrest when overexpressed in MCF7 cells [66]. The role of IGFBP_2_ cell cycle regulation has also been demonstrated. IGFBP_2_ expression has been found to modulate β-catenin (a key effector of cell cycle regulation) in an IGF-1R dependent manner. It has also been found to be associated with lymph node metastasis in BrCa cells [67]. In contrast, IGFBP_5_ induces cellular adhesion and inhibits migration of MCF7 cells via the activation of AKT in an IGF-independent manner, consistent with previous studies suggesting an apoptotic role for IGFBP_5_ in BrCa [68]. IGFBP_4_ may inhibit IGF-1-mediated effects, including tumor promoting ones, through the formation of IGF-1-binding complexes. Like all IGFBPs, IGFBP_4_ is sensitive to several proteases. Recombinant IGFBP_4_, capable of IGF-1 binding and resistant to protease cleavage by pregnancy-associated plasma protein-A (PAPP-A), has been shown to block mammary tumor growth and inhibit angiogenesis in a murine BrCa model [69].

### IGF-1 receptor (IGF-1R) in breast cancer

IGF-1R mediates the anti-apoptotic and tumorigenic effects of IGF-1 and, as expected, is frequently overexpressed in BrCa. Consequently, several studies have indicated a correlation of IGF-1R expression with disease development [70,71]. Women with high cytoplasmic levels of IGF-1R in epithelial cells found in benign terminal duct lobular units (TDLU’s) biopsies, have up to 15 times increased BrCa incidence [72].

Once cancer has been established, the importance of IGF-1R for disease progression remains unclear. Increased IGF-1R levels have been detected in many cases of breast malignancy, most often independently of cancer subtype, ER, PR or HER2 status. Furthermore, many studies indicate a down-regulation of IGF-1R upon cancer progression, whereas others report elevated levels in metastatic stages. Increased tissue IGF-1R mRNA levels strongly correlate with poor patient clinical outcomes across different molecular BrCa subtypes [73], whereas IGF-1R is highly expressed in patients with early BrCa and overall positively associated with good prognostic variables. IGF-1R may have differential prognostic impact in BrCa molecular subtypes; IGF-1R has been associated with favorable outcome in patients with the luminal B BrCa molecular subtype, in contrast to HER2 enriched patients [74]. Additionally, a positive association between IGF-1R and better clinical outcomes in hormone receptor positive, HER2 negative tumors was recently reported [75], while in another luminal subtype group, IGF-1R mRNA has been significantly correlated with improved BrCa-specific survival (BCSS). The same study found a correlation between IGF-1R protein levels and prolonged BCSS and association between IGF-1R mRNA expression and both relapse-free survival (RFS) and BCSS indicating a concurrence between IGF-1R mRNA and protein levels in primary breast tumors [76].

Several studies have attempted to clarify the relationship between specific genetic variants of *IGF-1R* and disease development and progression. Instead of typical functional polymorphisms in the coding region of *IGF-1R* gene, recent studies aimed to identify novel genetic variants located in untranslated regions (UTRs), such as miRNA’s binding sites, that could potentially regulate the expression patterns of *IGF-1R*. Although a wealth of evidence supports the key role of miRNAs in several tumorigenic processes, only a few studies have evaluated the potential association between polymorphisms in miRNA binding sites and cancers. The specific SNP (*rs28674628*) is located in a predicted binding site for the miRNA miR-515-5p, which directly regulates IGF-1R levels, in the 3′ UTR of the *IGF-1R* gene as demonstrated by computational analysis. Gilam et al. showed that the *rs28674628* is significantly associated with earlier age of diagnosis and increased BrCa risk among Jewish BRCA1 mutation carriers [77]. The *rs2016347* SNP is also located in the 3′ UTR of *IGF-1R* and *in silico* analysis predicted a functional role through transcriptional regulation and possibly miRNA binding. Indeed, patients carrying the G allele of the *IGF-1R rs2016347* polymorphism have poorer prognosis compared with those carrying G/T or T/T and has been associated with increased risk of tumor progression and death [78]. Seven others SNPs located in intron regions of the *IGF-1R* gene have been also associated with BrCa risk in Korean women [79]. Future studies may apply a Mendelian randomization approach to elucidate the association between functional *IGF-1R* polymorphisms and BrCa risk.

**Figure 2:**
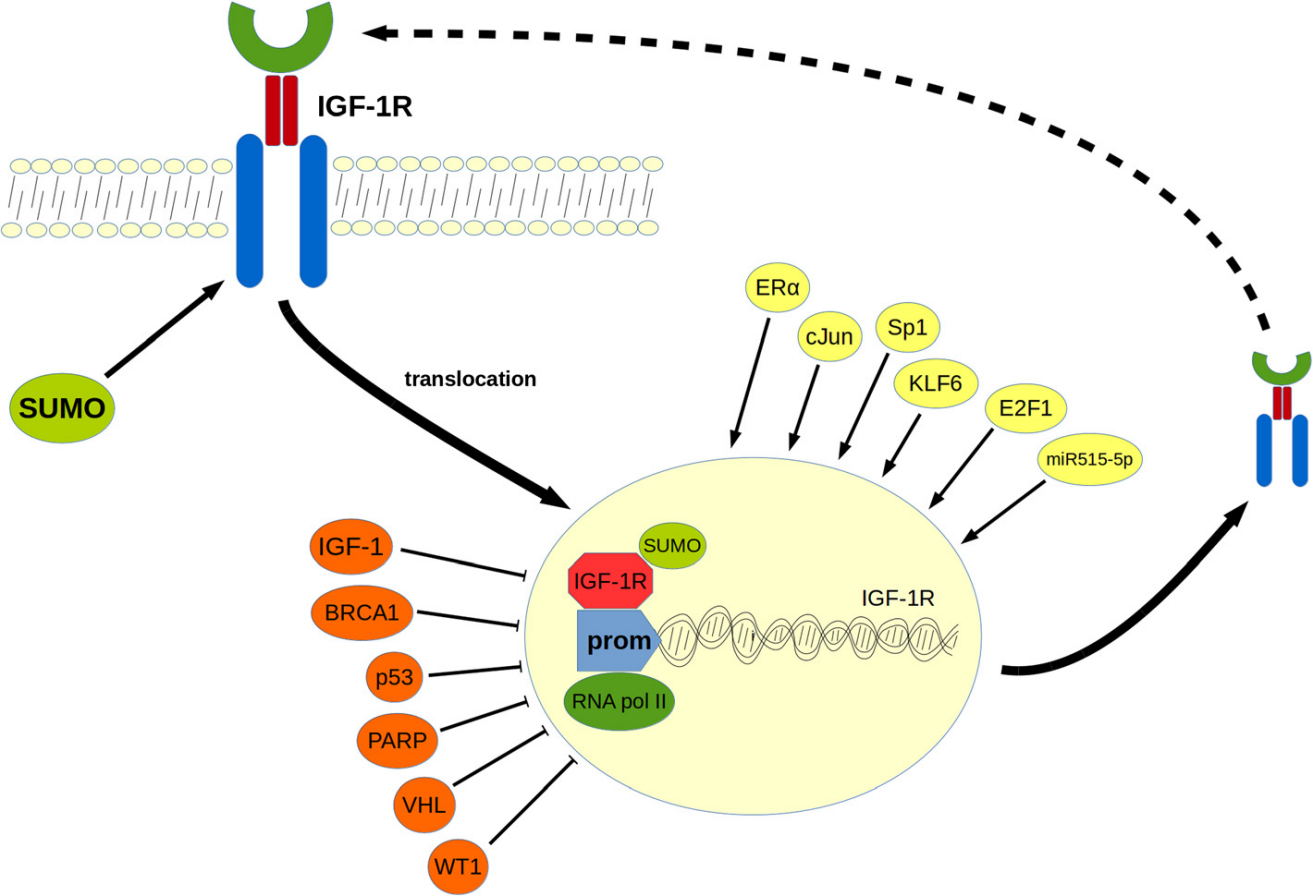
**The IGF-1 receptor (auto-) regulation mechanism in breast cancer.** SUMOylation of the IGF-1 receptor drives IGF-1R to nuclear translocation. Nuclear IGF-1R upregulates IGF-1R protein levels through direct binding to its own promoter. Other factors could also contribute to IGF-1R transcriptional regulation acting both as enhancers (ERα, miR515-5p and transcription factors c-Jun, Sp1, KLF6, E2F1,) or inhibitors (IGF-1, BRCA1, PARP, tumor suppressors p53, VHL, and transcription factor WT1). SUMO: small ubiquitin-like modifier; ERα: Estrogen receptor α; BRCA1: Breast cancer 1-early onset protein; KLF6: Kruppel-like factor 6; PARP: Poly-ADP ribose polymerase; VHL: von Hippel Lindau protein; WT1: Wilms tumor 1 protein.

Propelled by the data accumulated by miRNA approaches, researchers are now focusing on elucidating the molecular mechanisms underlying the gene regulation of *IGF-1R*. Recent studies have provided new insights into the molecular functions and biological significance of IGF-1R in BrCa by suggesting the role of novel transcription factors and mechanisms implicated in *IGF-IR* regulation. Regulation of *IGF-1R* is mainly achieved at the transcriptional level through transcription factors interfering with the GC-rich “initiator” motif (lacking TATA and CCAAT boxes) of the *IGF-1R* promoter in either a stimulatory or inhibitory fashion. Previous studies have reported a down regulation of *IGF-1R* potentiated by the cognate IGF-1 ligand. Several other molecules have been involved in these processes. These include molecules that normally inhibit *IGF-1R* expression such as the tumor suppressor proteins BRCA1, p53, the Wilm’s tumor (WT1) and von Hippel-Lindau (VHL), as well as molecules that stimulate IGF-1R production such as Sp1, KLF-6 and ERα [80-86]. Using a novel DNA affinity chromatography, Sarfstein et al. reported a total of 63 *IGF-1R*-promoter binding proteins (mostly nuclear) belonging to previously known or novel transcription factors as well as non-DNA-specific binding proteins involved in major cellular processes, including proliferation, apoptosis, protein synthesis, DNA repair, tumor suppression and oncogenesis. From these proteins, 24 have been shown to bind to the *IGF-1R* promoter only in MCF7 cells, whereas 19 only in C4.12.5 cells (ER-negative) and even those that are ubiquitous have been found to be expressed in different levels within these cells. This suggests a distinct *IGF-1R* regulation depending on ER status. *ChIP* assays and coexpression experiments have shown that PARP1, Sp1, c-jun and HMGA1 directly bind to *IGF-1R* promoter *in vivo* and c-jun, KLF6 and E2F1 enhance its activity [87]. Of note, two independent groups simultaneously reported in 2010 that IGF-1R undergoes nuclear translocation in human cancers, indicating that IGF-1R itself may act as a transcription factor [88,89]. Although IGF-1R lacks a nuclear localization sequence, experiments revealed that SUMOylation events as well as the receptor’s kinase activity are responsible for nuclear translocation. Co-localization with RNA-polymerase II and binding to chromatin, as shown by co-precipitation with histone H3, further support a transcriptional regulation role for IGF-1R [88,89]. Subsequently, a relevant novel autoregulation function for IGF-1R was reported [3] revealing that IGF-1R not only translocates to the nucleus but also specifically binds to its own promoter in ER^−^ BrCa cells. This enhances the activity of the promoter and thus IGF-1R acts as a transcriptional auto-activator [3]. These results delineate the complex *IGF-1R* autoregulation in BrCa (Figure 2). They have broadened our understanding of the molecular functions of IGF-1R in different disease stages and highlight the potential of IGF-1R targeting in patients with BrCa.

The significance of IGF-IR in BrCa is further supported by the observation that absent or severely reduced IGF-1R expression leads to non-viable MCF7 cells after a few tissue culture passages [90]. An important area for future research will be to clarify the role of IGF-1R expression and downstream signaling pathways in different BrCa molecular subtypes. Whereas basal-like tumors frequently have mutations of p53 gene and therefore induce *IGF-1R* transcriptional upregulation, claudin-low subtypes frequently underexpress IGF-1R and closely resemble mammary epithelial stem cells expressing many of the EMT markers. Indeed, studies in transgenic mice reveal that epithelial-specific overexpression of IGF-1R induces mammary tumors with basal-like characteristics whereas epithelial-specific down-regulation of IGF-1R produces tumor with a more claudin-low molecular profile [91]. The same team has previously reported that doxycycline-induced downregulation of IGF-1R in mammary epithelium using the same animal model induced tumor regression [92]. Furthermore, mouse xenograft experiments showed increased IGF-1R prosphorylation in CD45^−^/CD24^−^/CD44^+^ breast cancer stem cell (BCSC), while activation of the PI3K/AKT/mTOR pathway facilitated BCSC maintenance and increased their EMT phenotype indicating a regulatory role for the IGF-1R pathway in BrCa stem/progenitor cells [93].

It is well known that IGF-1R shares a high degree of homology with both A and B isoforms of the Insulin receptor (IR) [94]. Binding of insulin to IR leads to similar molecular events as IGF-1R activation including phosphorylation of IRS-1 and consequently activation of both AKT and MAPK. The role of IR and especially IRA as an alternative to IGF-1R signaling in BrCa is highlighted by studies addressing a regulatory effect of IR in mammary tumorigenesis [95], increased proliferation of BrCa cells in response to insulin [96] and upregulation of IRA isoforms in breast tumors [97]. Furthermore, IR can form hybrid receptors with IGF-1R: IGF-1R/IRB has higher affinity for IGF-1 whereas IGF-1R/IRA has higher affinity for IGF-2 [98] and is involved in cancer signaling pathways [99]. This relationship delineates the significance of IR and IGF-2 mediated signaling in BrCa via IGF-1R downstream activation. IRA generally has higher affinity for IGF-2 than IRB. The IGF-2 receptor binds, among other proteins, IGF-2 and is proposed to have tumor-suppressing effects in BrCa [100]. Alterations in IGF-2R levels due to genetic abnormalities in breast tumors may lead to modulation of BrCa tumorigenicity possibly via an IGF-1R depended manner [101]. Reduced levels of functional IGF-2R may lead to increased IGF-2-mediated IGF-1R signaling. The significance of IR signaling in physiological processes such as glucose regulation indicates that future research should focus on targeting both IR and IGF-2 alongside with IGF-1R in BrCa therapeutics, in a tissue specific manner.

Recent studies also indicate that IGR-1R may be used as a molecular target for BrCa imaging. In a panel comparing other potential imaging targets (EGFR, HER2, GLUT1 and others), IGF-1R was found to be suitable for molecular imaging strategies in 80% of female and 77% of male breast tumors [102,103].

### Estrogen receptor (ER) and the IGF-1 system in breast cancer

**Figure 3:**
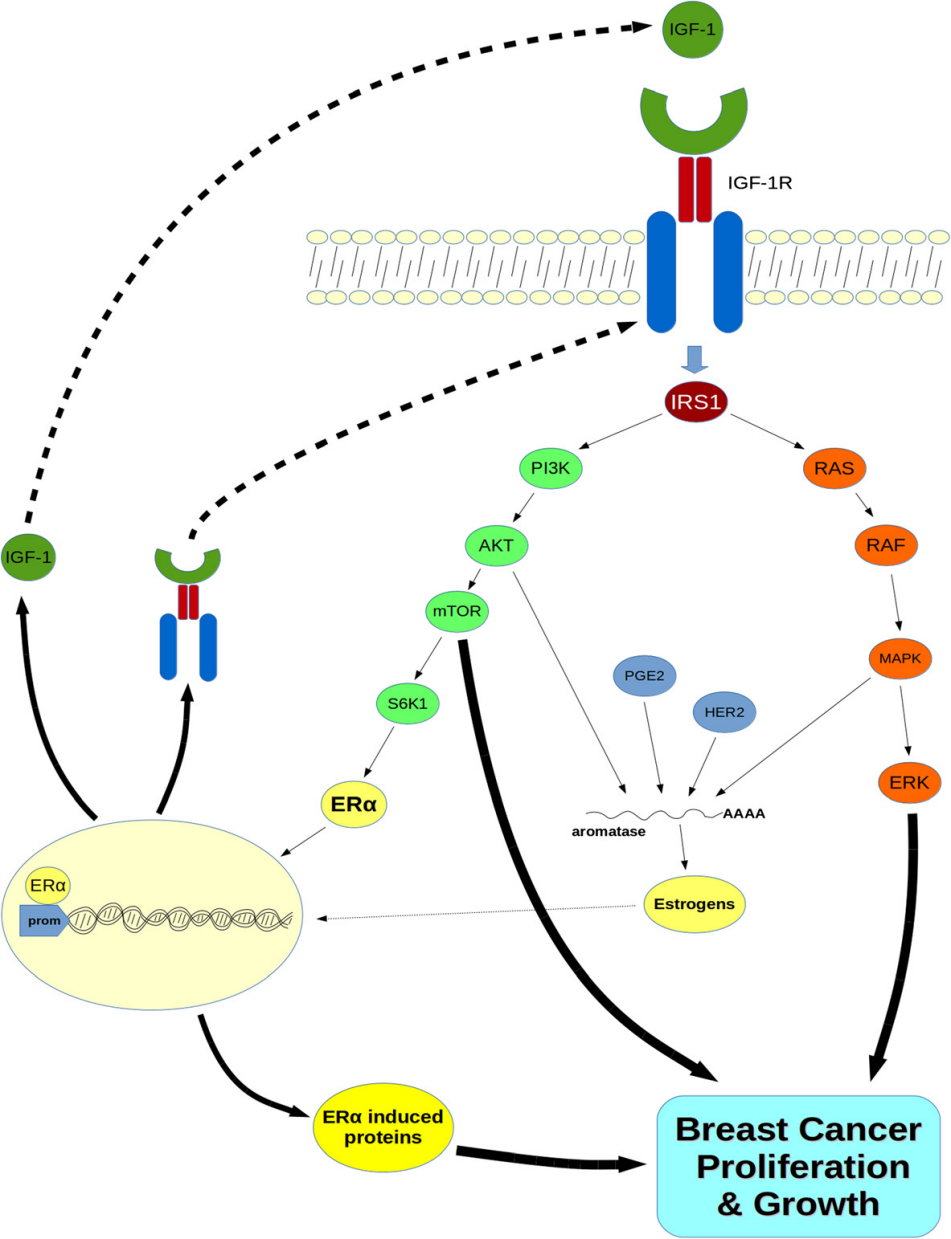
**Schematic representation of the crosstalk between the IGF-1 system and estrogens in breast cancer.** Binding of IGF-1 to IGF-1R triggers both PI3K/AKT/mTOR and MAPK/ERK signaling pathways, leading to enhanced breast cancer growth and proliferation. S6K1, a downstream molecule of the AKT/mTOR pathway induces phosphorylation of ERα. Activated ERα acts as transcription factor, through direct binding to promoter of target genes, consequently upregulating IGF-1, IGF-1R and other ERα-induced genes which also stimulate breast cancer progression. Prostaglandin E2 (PGE2) along with HER2 and both phosphorylated AKT and MAPK enhance aromatase activity through post-transcriptional mechanisms leading to elevated estrogen levels. Estrogens further contribute to transcriptional regulation of both IGF-1 and IGF-1R, consequently recycling the system’s signaling, enhancing growth and proliferation of breast tumors. S6K1: Ribosomal protein S6 kinase 1 protein; mTOR: mammalian target of rapamycin; HER2: human epidermal growth factor receptor 2 protein.

The importance of steroids and especially the ER status in disease progression is well established. Its significance is further supported by the fact that anti-estrogens, such as tamoxifen are routinely used in BrCa treatment. Crosstalk between growth factors and estrogens may be responsible for the development of estrogen-independent BrCa tumors [104]. Although the exact mechanism is not currently known, it is evident that there is a cross-talk between the IGF-1 system, ER and the cognate ER ligand 17β-estradiol (E2). BrCa cells have a differential response to IGF-1 with regards to both proliferation and survival depending on their ER status. Specifically, cells expressing both IGF-1R and ER demonstrate synergistic or additive growth effects in response to simultaneous administration of ligands (IGF-1, E2) [105]. Many components of the IGF-1 system are under the transcriptional control of ER/E2. A well-established example of this phenomenon is IGF-1R modulation by ERα as described above. A bi-directional regulation has also been proposed whereby IGF-1 induced pathways regulate ERα- dependent functions and *vice versa.* The mechanism underlying this cross-talk may be particularly important for the development of combination treatment strategies. Becker et al. demonstrated that the IGF-1/IGF-1R axis induces phosphorylation of ERa through ribosomal S6 kinase 1 (S6K1), a downstream molecule of the PI3K/AKT/mTOR pathway. Through chromatin and promoter binding, activated ERα modulates both the transcriptional regulation of ERα induced genes as well as BrCa cell growth and proliferation [4]. Other recent studies suggest that IGF-1 may be regulated by both ERα and BRCA1. IGF-1 expression has been shown to be activated by estrogens whereas breast tumors with BRCA1 mutations demonstrate elevated IGF-1 levels. Both ERα and BRCA1 can bind to an estrogen-responsive element like site (EREL) in the *IGF-1* promoter, thus enhancing or suppressing IGF-1 expression respectively [5]. Aromatase is a key enzyme during estrogen biosynthesis and is routinely used as a therapeutic target in ER positive BrCa tumors. Prostaglandin E2 (PGE2) along with HER2 and growth factors enhance aromatase activity via post-transcriptional mechanisms in BrCa cells mediated by the IGF-1/IGF-1R axis through both AKT and MAPK pathways. Thus, IGF-1 pathways can enhance aromatase activity via post-transcriptional modifications that do not affect aromatase protein levels [106]. The two subtypes of ER (ERα and ERβ) are found in different levels in breast tumors and can produce distinct cellular responses. However, we still lack a detailed understanding of the complex interaction between the ERα and ERβ subtypes with the IGF-1/IGF-1R axis and E2. MCF7 cells engineered to express reduced levels of IGF-1R demonstrate decreased proliferation and increased apoptosis in response to E2 compared to controls. The ERα/ERβ ratio is also impaired in these cells. Specifically, ERα is reduced whereas ERβ is elevated resulting in increased phosphorylation of p38 MAPK and activation of the p53 substrate protein, leading to apoptosis [90]. Other findings also support a cross-talk between E2 and the IGF-1/IGF-1R axis. It has been demonstrated that *in vitro* E2 and IGF-1 co-regulate a number of genes comprised mainly of tumor suppressing factors associated with poor disease outcome. E2 can also induce IGF-1R expression in mouse xenograft models [107,108]. Furthermore, IGF-1 alone in MCF7, or both IGF-1 and E2, in BrCa cells overexpressing the IGF-1R, can induce the IGF-1R/ERα association. IGF-1 stimulates activation of ERα, which consequently binds to IGF-1R, inducing downstream phosphorylation of AKT and ERK1/2 leading to increased cell growth via enhanced IGF-1-signaling pathways [109]. Tian et al., demonstrated that the IGF-1/ERα cross-talk is altered during development, and this may corroborate reports indicating an age-depended association between IGF-1 levels and BrCa risk. They proposed a non-genomic mechanism based in *in vivo* model study, in which ERα forms a complex with IRS1, triggering PI3K/AKT pathway in the prepubertal stage. This is in contrast to the postpubertal stage, where the reduced ERα levels prevent the ERα/IRS1 complex formation, and alter the signaling via the Raf/MAPK pathway [110]. Additionally, a non-genomic crosstalk between ERα and both Akt and ERK signaling pathways has been reported in obese postmenopausal women [111]. Although the exact mechanism is not clear, estrogens may be modulated by the IGF-1 system via both transcriptional and post-transcriptional mechanisms leading to increased BrCa proliferation and growth through activation of the IGF-1 signaling pathways (Figure 3).

### Interaction between the IGF-1 system and other growth factors in breast cancer

The IGF-1/IGF-1R axis may interact with other growth factors in the BrCa milieu. IGF-1 may cross-talk with the transforming growth factor beta-1 (TGFβ1), a molecule known to induce EMT, in BrCa. IGF-1 can activate matrix metalloproteinases through both AKT and MAPK pathways subsequently activating TGFβ1 leading to nuclear translocation of β-catenin and EMT [112]. Other studies also contribute to this crosstalk of IGF-1 with b-catenin and the Wnt pathway. Phyllodes tumors are composed of both stroma and epithelium and are considered to be rare breast malignancies. It has been suggested that IGF-1 alongside with Wnt signaling contributes to the β-catenin nuclear translocation found in phyllodes tumors [113]. Very recently, Liao et al., showed that IGF-1 induces MUC1 expression, a glycoprotein engaged in multiple cancer-related pathways, via AKT signaling, promoting translocation of β-catenin and EMT progression in MCF7 cells [114]. Thus, IGF-1, possibly through the involvement of other molecules, cross-talks with the Wnt/β-catenin pathway, via both AKT and/or ERK signaling, promoting migration of breast tumors. Other lines of evidence indicate an association with the vascular endothelial growth factor (VEGF)/ VEGFR axis, which comprise of key molecules for angio- and lymphangio- genesis as well as tumor (including breast) progression and metastasis. Morgillo et al.*,* indicate a significant association between high circulating levels of IGF-1, IGFBP_3_ and VEGFc with lymph node metastasis in endocrine-responsive BrCa patients [115]. Both ERK and AKT can contribute in IGF-1 induced upregulation of VEGF-c and may play important roles in lymph node metastasis of BrCa [116]. Furthermore, both IGF-1R and VEGFR-2 were found in a liquid biopsy assay to be co-expressed in circulating tumor cells (CTCs) [117]. Since Lu et al. demonstrated that increased IGF-1 levels are associated with trastuzumab resistance [118], recent focus has turned into clarifying the cross-talk between the IGF-1 system and the Epidermal Growth Factor Receptors (EGFR) family. ERBB2 (HER2) may contribute to decreased IGF-1R expression during mammary tumorigenesis [119]. An association between IGFBP and ERBB2 in IGF-1-dependent tumor transformation has been reported in mammary luminal epithelial cells [120]. Dearth et al., demostrated that ERBB2-induced mammary tumorigenesis is independent of circulating IGF-1 levels, indicating that the IGF-1/HER2 cross-talk may occur via autocrine and paracrine signaling in BrCa [121]. The IGF-1 system may also interact with components of the extracellular matrix (ECM) affecting tumor growth and progression. Complexes of IGF-1 with IGFBP3 or IGFBP5 and vitronectin (VN) have been shown to induce MCF7 survival and regulation of genes implicated in migration and invasion processes [122].

### Targeting the IGF-1R in breast cancer: clinical evidence

Both *in vitro* and *in vivo* evidence strongly suggest that the IGF-1 system could serve as a promising target candidate in BrCa therapeutics. Indeed, over the last decade, many strategies targeting several components of the IGF-1 system, have been tested. The majority of these involved targeting the IGF-1R, either with monoclonal antibodies (mAbs) causing internalization of the receptor, or by blocking the receptor’s tyrosine kinase domain activation using receptor tyrosine kinase inhibitors (RTKIs). The first recruited mAb against IGF-1R is CP-751,871 (figitumumab) which has demostrated good pre-clinical antitumor efficacy against several cancers including BrCa [123]. Only mild toxicities induced by figitumumab have been reported [124]. Figutumumab has also been tested in phase I (NCT00635245 and NCT01536145) and phase II (NCT00372996) clinical trials against BrCa with apparently negative unpublished results. IMC-A12 (cixutumumab) is another mAb against IGF-1R. It has been tested in a phase I trial in combination with temsirolimus in patients with resistant ER positive breast tumors and only mild toxicities were seen [125]. A phase II trial of cixutumumab is currently ongoing (NCT00699491). Another phase II trial (NCT00684983) is evaluating the effect of cixutumumab in combination with capecitabine and lapanitib in patients with metastatic HER2+ BrCa [126]. R1507 (RO4858696) is another monoclonal IgG antibody specifically targeting IGF-1R that has demonstrated no dose-limiting toxicities [127]. A phase Ib study showed that R1507 is well tolerated when added to six standard chemotherapy regiments in patients with various solid tumors, including BrCa [128]. Another phase I trial (NCT00882674) in women with operable breast cancer and a phase II trial (NCT00796107) of R1507 in combination with letrozole in women with advanced breast tumors have finished, with results pending publication. The same mAb has also successfully been used for imaging breast cancer *in vivo* using either SPECT and/or PET imaging techniques [129]. Unlike most of the mAbs against IGF-1R, figutumumab and SCH717454 have the ability to block signals induced by hybrid receptors [124,130] and this regimen has been tested in a phase II clinical trial (NCT00954512) in patients with advanced solid tumors. The reported adverse events common to most IGF-1R mAbs include anemia, fever, diarrhea, arthralgia, leukopenia, thrombocytopenia, rash, fatigue and anorexia with hyperglycemia being the most common. Noticeable, hyperglycemia was not reported for R1507 [128].

Since the first RTKI (NVP-ADW742) targeting IGF-1R [131], research efforts have focused in developing of these molecules as potential IGF-1R inhibitors. Most of these molecules target the tyrosine domain of both IGF-1R or IR, which may compromise specificity. Although many of these molecules have demonstrated good preclinical results, only a few have actually been translated in ongoing clinical trials, probably due to increased cytotoxicity events. Indeed, production of the NVP-ADW742 was halted due to toxicity issues. OSI-906 (linsitinib), a RTKI targeting both IGF-1R and IR, was investigated as a treatment for hormone sensitive breast cancer in combination with endocrine therapy and erlonitib in a phase II clinical trial (NCT01205685). Unfortunately, this study was terminated as all patients experienced severe toxicities and tumor progression. BMS-754807, another dual RTKI is being tested in a currently ongoing phase II trial (NCT01225172) in combination with letrozole in women with andvanced ER positive, non-steroidal aromatase inhibitor resistant breast tumors [132]. Another phase I/II trial of BMS-754807 in combination with transtuzumab in patients with advanced or metastatic HER2 positive breast tumors has been completed (NCT00788333) and complete results remain to be published. Except specificity another limitation of the RTKI approach is, in contrast to mAbs, the lack of receptor internalization, which means that IGF-1R could retain it’s activity a while after inhibition treatment ends. Targeting the IGF-1 system via inhibiting IGF-1R signal transduction cascade presents limitations. The complexity of this system, eliminates the possibilities for efficient targeting. Even if we could specifically block the IGF-1R and not IR, unwanted effects may arise such as glucose dysregulation and diabetes due to the inhibition of hybrid IGF-1R/IR receptors. On the other hand, targeting of hybrid receptors in BrCa therapeutics, could be desirable as they may act more as IGF-1R rather than IR. In addition, IGF-1R targeting may not inhibit the IGF-2 mediated signal transduction via IR and/or hybrid receptors. Further insights into this complex crosstalk, may help us overcome some disappointing clinical results and allow the translation of novel molecules and strategies to specifically suppress the IGF-1R-induced tumor promoting effects.

## Conclusions

A growing body of evidence supports the association of the IGF-1 system with BrCa establishment and progression. Conflicting results may arise from discordant methodologicals approaches, distinct molecular subtypes studied, genetic differences between different populations and tumor heterogeneity.

The complex IGF-1/IGF-1R axis cascade has received significant attention to date and a wealth of experimental evidence from both *in vitro* and *in vivo* models and human studies have implicated the IGF-1 system with BrCa biology. The prospect of efficiently targeting the IGF-1 system in BrCa is certainly attractive. Further insight on the molecular mechanisms driving the disease via the IGF-1 system will open new avenues for the diagnosis and treatment of BrCa. Numerous challenges will have to be overcome prior to reaching the goal of IGF-1 regulation for the prevention and management of BrCa. Elucidating the IGF-1 expression patterns and diverse molecular pathways may allow the development of effective diagnostic and treatment strategies against BrCa that may prove beneficial for selective population subgroups.

### Ethical approval

Reported research carried on humans are in compliance with the Helsinki Declaration, whereas experimental research on animals follow internationally recognized guidelines.

## Abbreviations

IGF-1: Insulin-like growth factor-1
GFBP_1–5_: Insulin-like growth factor binding proteins 1–5
BrCa: Breast cancer
GH: Growth hormone
IRS-1: Insulin receptor substrate 1
PI3K: Phosphatidylinositol 3-kinase
MAPK: Mitogen-activated protein kinase
ERα-β: Estrogen receptor α-β
PR: Progesterone receptor
SUMO: Small ubiquitin-like modifier
BRCA1: Breast cancer 1-early onset protein
KLF6: Kruppel-like factor 6
PARP1: Poly-ADP ribose polymerase 1
VHL: Von hippel lindau protein
WT1: Wilms tumor 1 protein
S6K1: Ribosomal protein S6 kinase 1 protein
mTOR: Mammalian target of rapamyci
HER2: Human epidermal growth factor receptor 2 protein
SNP: Single-nucleotide polymorphism
PBS: Phosphate buffer saline
EMT: Epithelial to mesenchymal transition
CTCs: Circulating tumor cells
ECM: Extracellular matrix
BD: Binding domain
VEGFc: Vascular endothelial growth factor receptor c
EGFR: Epidermal growth factor receptor
TGFβ: Transforming growth factor β
UTR’s: Untranslated regions
EREL: Estrogen-responsive element-like site
PGE2: Prostaglandin E2
ChIP: Chromatin immunoprecipitation
COX-2: Cyclooxygenase-2
VN: Vitronectin
ECR: Evolutionary conserved regions
DFS: Disease-free survival
TDLU's: Terminal duct lobular units
BCSC: Breast cancer stem cell
BCSS: BrCa-specific survival
mAb: Monoclonal antibody
RTKI: Receptor tyrosine kinase inhibitor
IR: Insulin receptor
